# Mol* Volumes and Segmentations: visualization and interpretation of cell imaging data alongside macromolecular structure data and biological annotations

**DOI:** 10.1093/nar/gkad411

**Published:** 2023-05-17

**Authors:** Aliaksei Chareshneu, Adam Midlik, Crina-Maria Ionescu, Alexander Rose, Vladimír Horský, Alessio Cantara, Radka Svobodová, Karel Berka, David Sehnal

**Affiliations:** National Centre for Biomolecular Research, Faculty of Science, Masaryk University, 625 00 Brno, Czech Republic; National Centre for Biomolecular Research, Faculty of Science, Masaryk University, 625 00 Brno, Czech Republic; Biological Data Management and Analysis Core Facility, Centre for Structural Biology, CEITEC – Central European Institute of Technology, Masaryk University, 625 00 Brno, Czech Republic; National Centre for Biomolecular Research, Faculty of Science, Masaryk University, 625 00 Brno, Czech Republic; Mol* Consortium, San Diego, CA 92109, USA; National Centre for Biomolecular Research, Faculty of Science, Masaryk University, 625 00 Brno, Czech Republic; Biological Data Management and Analysis Core Facility, Centre for Structural Biology, CEITEC – Central European Institute of Technology, Masaryk University, 625 00 Brno, Czech Republic; National Centre for Biomolecular Research, Faculty of Science, Masaryk University, 625 00 Brno, Czech Republic; Biological Data Management and Analysis Core Facility, Centre for Structural Biology, CEITEC – Central European Institute of Technology, Masaryk University, 625 00 Brno, Czech Republic; National Centre for Biomolecular Research, Faculty of Science, Masaryk University, 625 00 Brno, Czech Republic; Biological Data Management and Analysis Core Facility, Centre for Structural Biology, CEITEC – Central European Institute of Technology, Masaryk University, 625 00 Brno, Czech Republic; Department of Physical Chemistry, Faculty of Science, Palacký University Olomouc, 779 00 Olomouc, Czech Republic; National Centre for Biomolecular Research, Faculty of Science, Masaryk University, 625 00 Brno, Czech Republic; Biological Data Management and Analysis Core Facility, Centre for Structural Biology, CEITEC – Central European Institute of Technology, Masaryk University, 625 00 Brno, Czech Republic

## Abstract

Segmentation helps interpret imaging data in a biological context. With the development of powerful tools for automated segmentation, public repositories for imaging data have added support for sharing and visualizing segmentations, creating the need for interactive web-based visualization of 3D volume segmentations. To address the ongoing challenge of integrating and visualizing multimodal data, we developed Mol* Volumes and Segmentations (Mol*VS), which enables the interactive, web-based visualization of cellular imaging data supported by macromolecular data and biological annotations. Mol*VS is fully integrated into Mol* Viewer, which is already used for visualization by several public repositories. All EMDB and EMPIAR entries with segmentation datasets are accessible via Mol*VS, which supports the visualization of data from a wide range of electron and light microscopy experiments. Additionally, users can run a local instance of Mol*VS to visualize and share custom datasets in generic or application-specific formats including volumes in .ccp4, .mrc, and .map, and segmentations in EMDB-SFF .hff, Amira .am, iMod .mod, and Segger .seg. Mol*VS is open source and freely available at https://molstarvolseg.ncbr.muni.cz/.

## Graphical Abstract

**Graphical Abstract:**
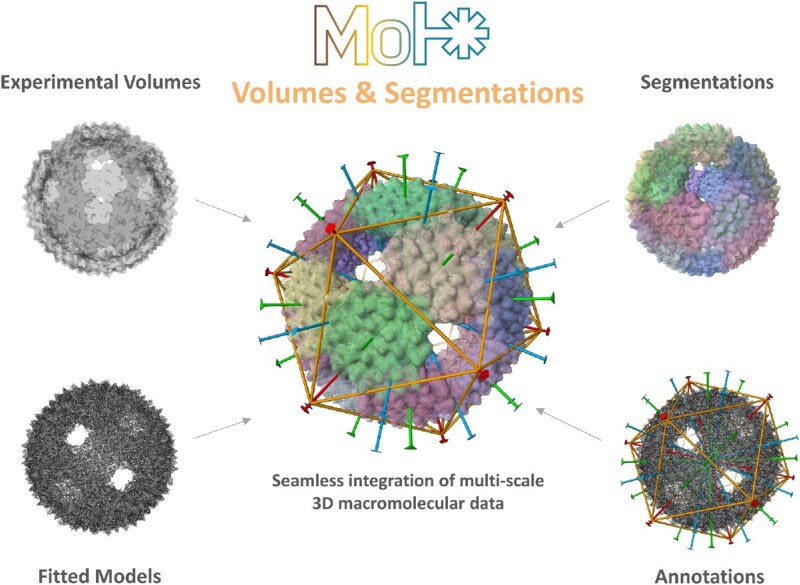

## INTRODUCTION

Segmentation, which is the decomposition of a two-dimensional (2D) or three-dimensional (3D) image into regions that can be associated with defined objects, has been recognized as the bridge to interpreting microscopy data in a biological context. While medical imaging has traditionally relied heavily on manual segmentation, advancements in segmentation tools (1,2), data format definition (3), and data sharing pipelines (4) have led to a substantial increase in the number of depositions of volume segmentation data from cell and molecular imaging experiments to public repositories (5,6). Whereas many desktop applications provide segmentation functionality and visualization options (7–12), there is very limited support for the visualization of 3D volume segmentations in public repositories.

For example, the Cell Image Library (CIL) (13) has integrated CDeep3M (11) for performing segmentation tasks on CIL entries, but it only provides visualization of 2D slices. The Brain Observatory Storage Service and Database (BossDB) (14) has integrated a Neuroglancer interface (https://github.com/google/neuroglancer) that facilitates seamless zooming through superimposed 2D slices, but does not allow the real-time examination of 3D volumes. Similarly, the Electron Microscopy Database (EMDB) (15) provides 3D visualization of densities, but segments are only defined on the 2D slices. Other repositories such as Electron Microscopy Public Image Archive (EMPIAR) (6) and Image Data Resources (IDR) (16) do not provide 3D visualization. Therefore, there is a need for accessible, web-based visualization of 3D volume segmentations, especially in the context of complex structural data and annotations linked across different databases.

We previously introduced Mol* as a library of tools for the visualization and analysis of macromolecular data (17). Mol* has become a large collaborative project, and its associated web-based 3D viewer Mol* Viewer (18) has been fully incorporated into the public interfaces of PDBe (19), RCSB PDB (20), and AlphaFold Protein Structure Database (21), enabling real-time visualization and interrogation of 3D models and related macromolecular data for millions of users.

Here, we introduce Mol* Volumes & Segmentations (Mol*VS) (https://molstarvolseg.ncbr.muni.cz/), a free tool based on Mol* and dedicated to the real-time visualization of large-scale volumetric data from cryo-EM, light microscopy, volume-EM, and other imaging experiments, as well as their segmentations and annotations for biological context. Mol*VS provides seamless access to all curated segmentation datasets available in EMDB and EMPIAR. Additionally, Mol*VS can be run locally to support the visualization and sharing of custom datasets containing volumetric segmentation data in several formats. Mol*VS is an open-source project hosted on GitHub (https://github.com/molstar/molstar-volseg).

## DESCRIPTION OF THE WEB SERVER

### Functionality

- Reading volumetric and segmentation data from public repositories (e.g. EMBD, EMPIAR).
- Processing input in formats used by public repositories, including EMDB-SFF .hff (https://www.ebi.ac.uk/emdb/documentation#seg-_model), 3D volume maps .ccp4, .mrc, and .map (https://www.ebi.ac.uk/emdb/documentation), and experimental support for OME-NGFF (22).
- Processing input in application-specific formats, including Amira .am (23), iMod .mod (24), and Segger .seg (25).
- Visualizing large-scale volumetric data, as well as volume and mesh segmentation data from various types of experiments including cryo-EM, light microscopy, volume-EM, and tomography.
- Displaying biological context annotations for each volume or mesh segment.
- Supplementing the volumetric and segmentation data with macromolecular coordinates.
- Concomitant display of different segmentations of the same dataset to facilitate visual comparison.
- Streaming data according to visualization needs.

Instructions on how to use Mol*VS are available at its web page.

### Architecture and implementation

**Figure 1.**
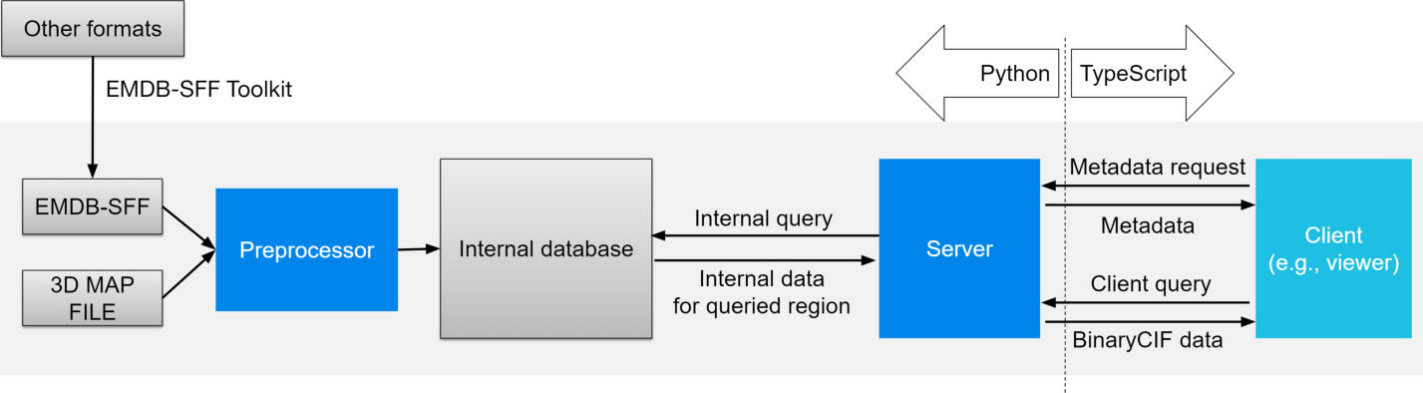
Mol*VS architecture and workflow.

Mol*VS processes volumetric and segmentation data and delivers it to a dedicated Mol* Viewer VS extension, so that even very large datasets can be visualized with low latency. Mol*VS has four major components, namely a preprocessor module (written in Python), an internal database with preprocessed data, a server module that queries the internal database (written in Python), and a client module (written in Typescript) that requests and interprets the data received so that it can be displayed (Figure 1).

### Workflow

The Mol*VS workflow (Figure 1) is not exposed to users but is designed to ensure seamless integration within the Mol* environment, effectively providing web-based concomitant and interactive visualization of 3D volumes of cells, organelles, and molecules, together with volume segmentations and their annotations, irrespective of the size of the original dataset.

#### Input processing

is only needed when the internal database has to be updated by adding, removing, or changing entries. By default, the *preprocessor* module of Mol*VS takes two inputs for each entry: a volume or mesh segmentation file in EMDB-SFF format (.hff) and a 3D map file (.map, .mrc, .ccp4) from the EM reconstruction. Segmentation input can also be provided in other formats (Amira .am, iMod .mod, Segger .seg), which will be internally converted to EMDB-SFF using the EMDB-SFF Toolkit (https://sfftk.readthedocs.io/en/latest/toolkit.html) integrated in Mol*VS. While the default workflow is optimized for EM data, Mol*VS also has experimental support for OME-NGFF input to facilitate the visualization of light microscopy data (not mentioned in Figure 1). The *preprocessor* module of Mol*VS converts the input into an internal format (Zarr, https://zarr.readthedocs.io), which is essentially a set of chunked, compressed, *N*-dimensional arrays. These preprocessed data are stored in the Mol*VS *internal database* in both original and downsampled forms, together with precomputed statistics, metadata, and internal biological annotations for each dataset (in JSON format).

#### Data delivery

Whenever data are requested by the Mol*VS *client* module, the *server* module performs a metadata request of the *internal database* and sends metadata to the *client*. Metadata is then used by the client to prepare the query for volumetric and segmentation data. Then, the *client* module sends the appropriate query for volumetric and segmentation data, specifying the volume/segmentation region and maximum acceptable size of the data. The *server* module decides the appropriate downsampling level based on the *client* request, queries the *internal database*, packs the requested volumetric and segmentation data into BinaryCIF format (26), and delivers them back to the *client* module.

#### Visualization

**Figure 2.**
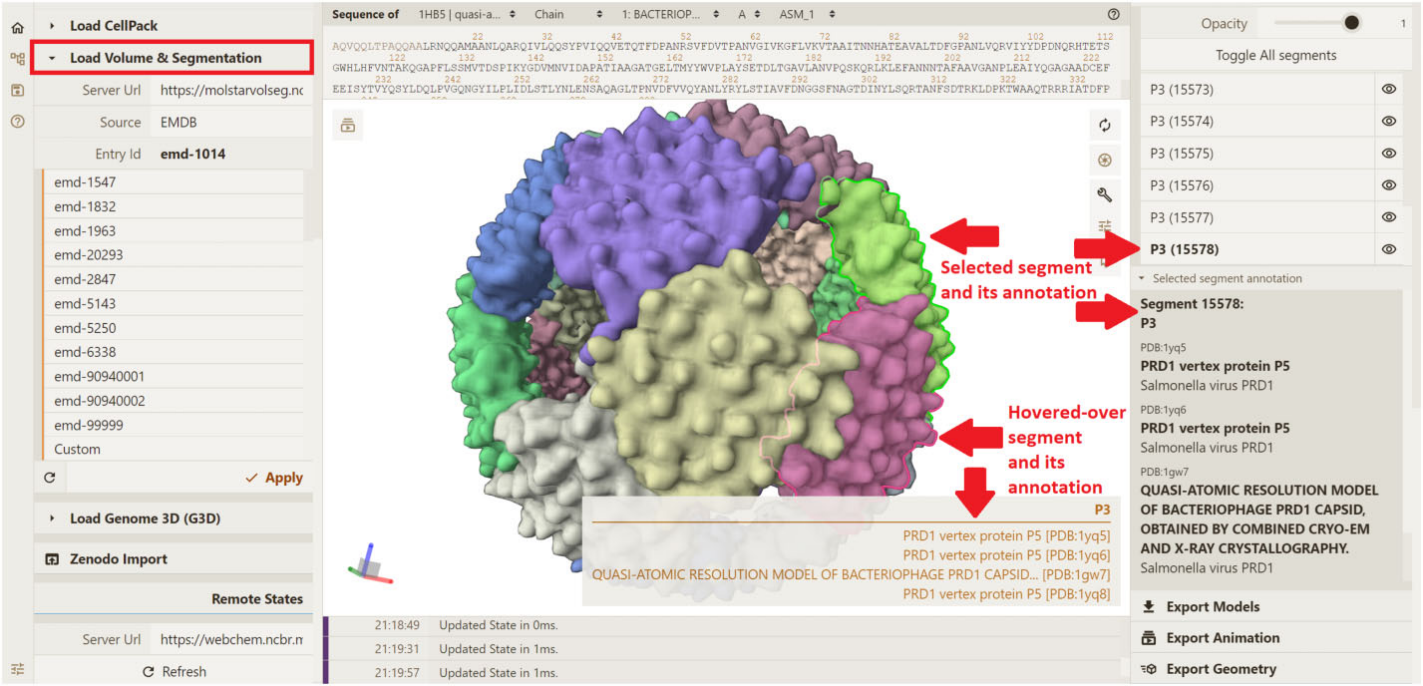
Visualization of segmentation data via Mol*VS. The VS extension can be accessed through the Load Volume & Segmentation tab in the Mol* Viewer UI. Segment annotations are shown both in the UI and in the 3D scene.

**Figure 3.**
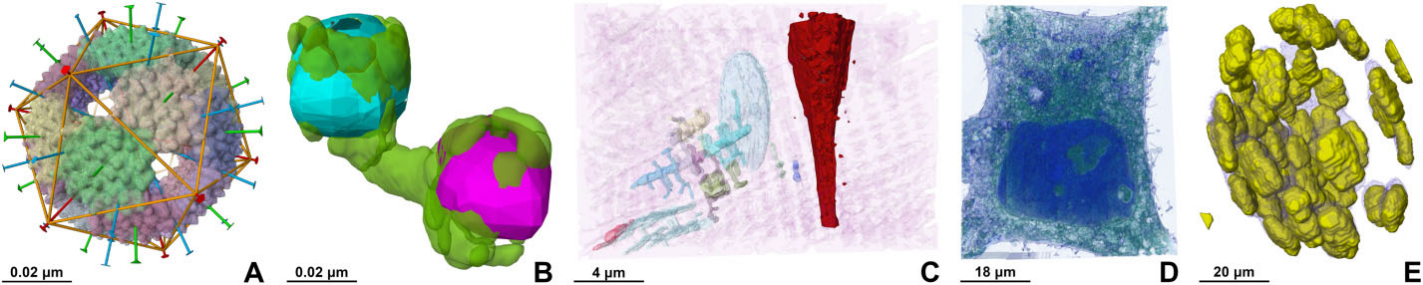
Showcase of Mol*VS visualization capabilities ranging from atomic to cellular scale. (**A**) 3D reconstruction of a large bacteriophage based on cryo-EM and crystallographic data with assembly symmetry axes, where the segmentation distinguishes macromolecular assemblies of its major capsid protein (EMD-1014 (27)). (**B**) Concomitant display of two segmentation outcomes (manual and automated) for a dataset from cryo-EM combined with individual-particle electron tomography imaging of human plasma lipoproteins (in purple and azure) in complex with a monoclonal antibody (in green) (EMD-9094 (28)). (**C**) Mitochondrial reticulum in murine skeletal muscle imaged using ion beam scanning EM, where segmentation distinguishes mitochondria from other cellular structures (e.g., blood vessels highlighted in dark red) (EMPIAR-10070 (29)). (**D**) HeLa cells imaged using confocal microscopy (EMPIAR-10819, not published yet). In the image, the cell and background is highlighted in dark blue while the surrounding environment is green. (**E**) Nuclear segmentation of mouse blastocysts imaged using confocal microscopy (image 6001240 from dataset idr0062 (30)).

is handled by the *client* module (the Mol* Viewer VS extension). Upon receiving data from the *server* module, the Mol* Viewer VS extension unpacks it, allowing Mol* Viewer to create a state tree with volume and segmentation data. The corresponding entities are rendered on the 3D canvas (Figure 2). Segment annotations are displayed via the same mechanisms that support annotations for amino acid residues and protein chains. The concomitant display of related entries from different public repositories effectively allows different types of data to be examined within the same biological context (Figure 3A). Different segmentations of the same dataset are stored in separate entries of the Mol*VS internal database, which can be displayed concomitantly to facilitate comparison (Figure 3B).

### Local instance of Mol*VS

While visualization is the focus of most end users, other categories of users can also benefit from Mol*VS. In particular, institutes and consortia who wish to share volume or mesh segmentation data with a private or public user community can host a local instance of Mol*VS. This way, their users can visualize the data in a web browser, without having to download anything. A step-by-step tutorial with all technical information needed to host Mol*VS locally is available in its GitHub repository.

## RESULTS AND DISCUSSION

Mol*VS can visualize volumes and segmentations obtained by a wide variety of imaging techniques and spanning resolutions from the atomic to cellular scale. Due to this versatility of Mol*VS, the outcomes of complex inter-disciplinary experiments coordinated across multiple research groups can now be examined effortlessly and interactively in a web browser. Several examples (Figure 3) are available for interactive visualization on the Mol*VS web page, with full explanations in the Mol*VS documentation (see Mol*VS web page).

### Database coverage

The Mol*VS internal database covers all EMDB and EMPIAR entries with segmentation data, and is updated periodically. Additionally, we provide entries derived from EMDB, BioImage Archive, and IDR datasets to showcase its support for application-specific segmentation formats and facilitate comparison. A full description of the contents of the internal database is available in the Mol*VS documentation (see its web page). Individual users and platforms providing access to cell imaging data can freely host a local instance of Mol*VS and fill the internal database with any content supported by the preprocessor module.

### Limitations and outlook

While recent years have seen an increase in the deposition of volume segmentation data, this trend is still in its infancy. Therefore, some of the source data may contain errors or are not well visualized by Mol*VS when default settings are applied. Users can resolve or mitigate such issues by following the instructions listed in the documentation (see Mol*VS web page), and can report issues or give suggestions via the Mol*VS GitHub repository. Furthermore, Mol*VS currently provides only experimental support for the OME-NGFF format, as showcased in entry idr-6001240 of the internal database (Figure 3E), because this format is still in active development.

Limitations related to source data availability and format standardization affect our ability to optimize Mol*VS at this time. Nonetheless, we believe that EMDB-SFF and OME-NGFF will become the standard formats for sharing and storing EM and light microscopy data, respectively. Therefore, we are fully committed to adjusting and extending the Mol*VS support for these formats as needed. In fact, we are actively cooperating with teams from EMDB, EMPIAR and BioImage Archive (31) to ensure that Mol*VS always contains the latest segmentation data available in these primary sources, and that all updates to the data format are adequately supported. We are confident that, by facilitating the web-based visualization of segmentation data and annotations, Mol*VS will promote the deposition of segmentation data in public repositories.

## CONCLUSION

Mol*VS (https://molstarvolseg.ncbr.muni.cz/) is a powerful web application for the interactive visualization of volumetric and segmentation data supported by macromolecular data and biological annotations. Volume data may originate from various imaging experiments, from cryo-EM to classical light microscopy. Segmentation data may be provided in generic (EMDB-SFF .hff, OME-NGFF) or application-specific formats (Amira .am, iMod .mod, or Segger .seg). Both volumetric and mesh segmentations are supported. Multiple segmentations for the same dataset can be compared easily. Data streaming allows interactive visualization, irrespective of the size of the original data set. Mol*VS facilitates the visualization of all EMDB and EMPIAR entries with segmentation data, but users may also run a local instance of Mol*VS to visualize and share custom datasets.

## DATA AVAILABILITY

The Mol*VS web server and its documentation are accessible at https://molstarvolseg.ncbr.muni.cz/. The current version of the source code is in the Supplementary Data, while the most recent version is available at https://github.com/molstar/molstar-volseg.

## Supplementary Material

gkad411_Supplemental_Files
