# Supplementary material for: Mol* Volumes and Segmentations: visualization and interpretation of cell imaging data alongside macromolecular structure data and biological annotations
Source: Nucleic Acids Res. 2023 May 17;51(W1):W326–30. doi: 10.1093/nar/gkad411 (PMC10320116; doi:10.1093/nar/gkad411)
Supplement: gkad411_Supplemental_Files [file gkad411_supplemental_files.zip › molstar-volseg-master/frontend/public/index.html]

Mol\* Volumes & Segmentations


You need to enable JavaScript to run this app.
