# Supplementary material for: Mol* Volumes and Segmentations: visualization and interpretation of cell imaging data alongside macromolecular structure data and biological annotations
Source: Nucleic Acids Res. 2023 May 17;51(W1):W326–30. doi: 10.1093/nar/gkad411 (PMC10320116; doi:10.1093/nar/gkad411)
Supplement: gkad411_Supplemental_Files [file gkad411_supplemental_files.zip › molstar-volseg-master/frontend/public/terms-of-use.html]

Mol\* Terms of Use


# Mol\* Terms of Use

1. We promote open science through its mission to provide freely available online services, database and software relating to data contributed from life science experiments to the largest possible community. Where we present scientific data generated by others we impose no additional restriction on the use of the contributed data than those provided by the data owner.
2. Attribution is expected (e.g. in publications, services or products) for any of our online services, databases or software in accordance with good scientific practice.
3. Any feedback provided to us on our online services will be treated as non-confidential unless the individual or organisation providing the feedback states otherwise.
4. We are not liable to you or third parties claiming through you, for any loss or damage.
5. We do not store personal data of any kind. We may make information about the total volume of usage of particular software or data available to the public and third party organisations who supply the software or databases without details of any individual’s use.
6. While we will retain our commitment to OpenScience, we reserve the right to update these Terms of Use at any time. When alterations are inevitable, we will attempt to give reasonable notice of any changes by placing a notice on our website, but you may wish to check each time you use the website. The date of the most recent revision will appear on this, the ‘Webchem Terms of Use’ page. If you do not agree to these changes, please do not continue to use our online services. We will also make available an archived copy of the previous Terms of Use for comparison.

---

# General Data Protection Regulation (GDPR)

Dear visitors, with respect to the “Regulation (EU) 2016/679 of the European Parliament and of the Council of 27 April 2016 on the protection of natural persons with regard to the processing of personal data and on the free movement of such data, and repealing Directive 95/46/EC (General Data Protection Regulation)” hereinafter GDPR we would like to inform you how your personal data are processed and secured.

Our website uses cookies. Cookies are text files that are stored in or by the Internet browser on the user’s computer system. If a user accesses a website, a cookie can be stored on the user’s operating system. This cookie contains a characteristic sequence of characters that allow the browser to be identified when accessing the website again.We use cookies to make our website more user-friendly. Cookies cannot be used for identifying visitors or abuse of login data. Most of the web browsers accepts cookies unless stated otherwise. By using these web pages you agree on storing cookies in your computer. The use of cookies can be restricted or canceled in your web browser settings.

Our web pages also use Google Analytics, which are solely used for statistical purposes. Remarketing and advertising services are deactivated by the administrator.

Thank you for understanding, our main goal is to help you to carry out your research in accordance with the personal data protection regulation.
