# Supplementary figures and images for: Mol* Volumes and Segmentations: visualization and interpretation of cell imaging data alongside macromolecular structure data and biological annotations
Source: Nucleic Acids Res. 2023 May 17;51(W1):W326–30. doi: 10.1093/nar/gkad411 (PMC10320116; doi:10.1093/nar/gkad411)

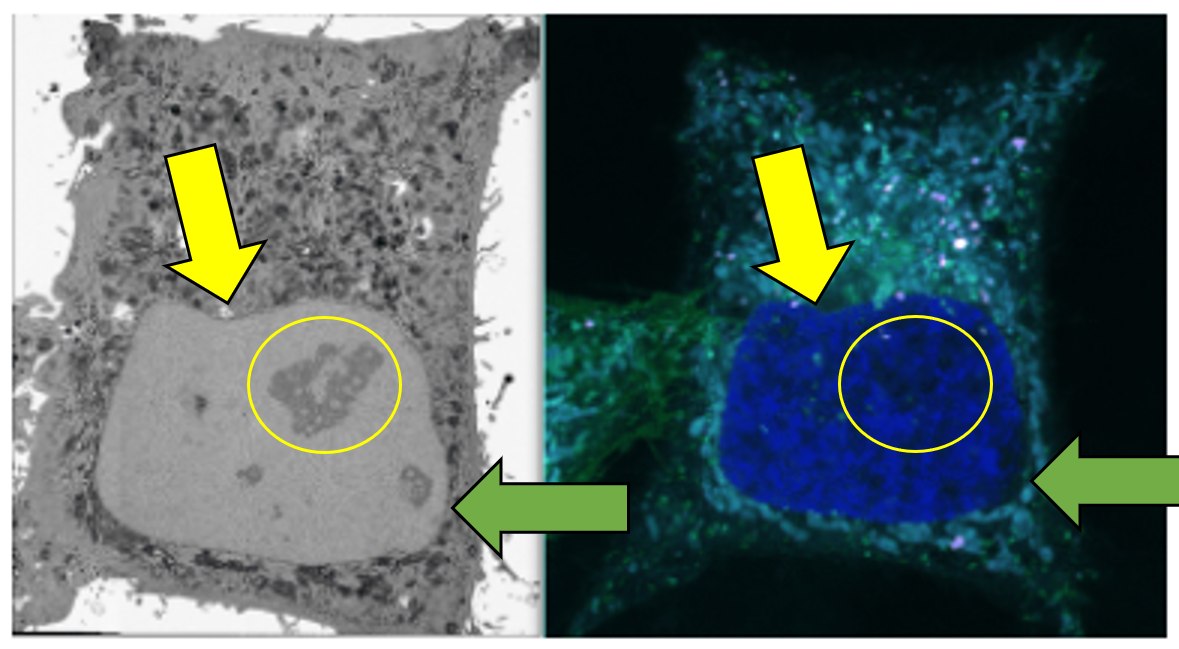

Supplement: gkad411_Supplemental_Files [file gkad411_supplemental_files.zip › molstar-volseg-master/frontend/public/emd-99999.png]

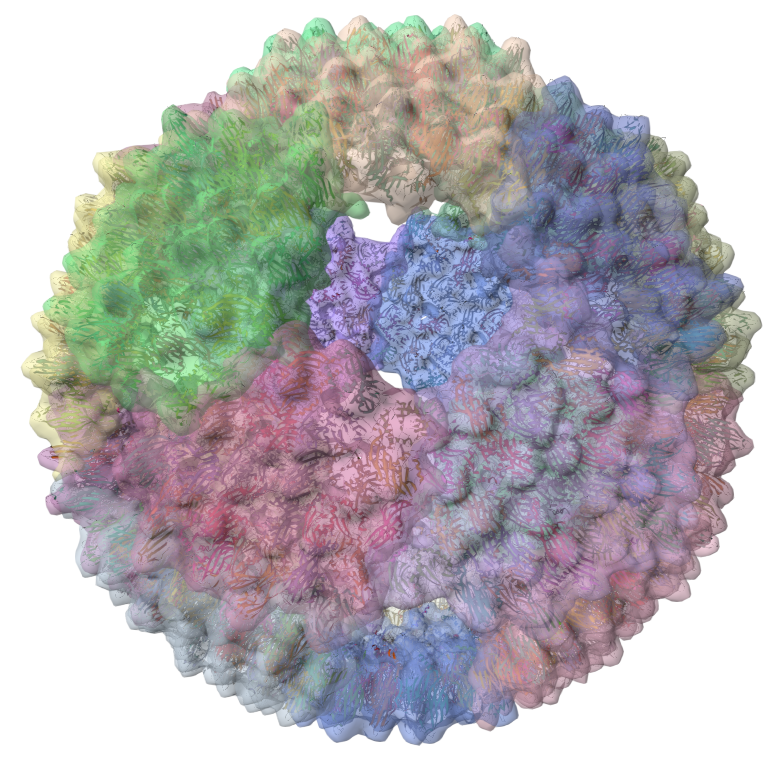

Supplement: gkad411_Supplemental_Files [file gkad411_supplemental_files.zip › molstar-volseg-master/frontend/public/img/examples/emd-1014.png]

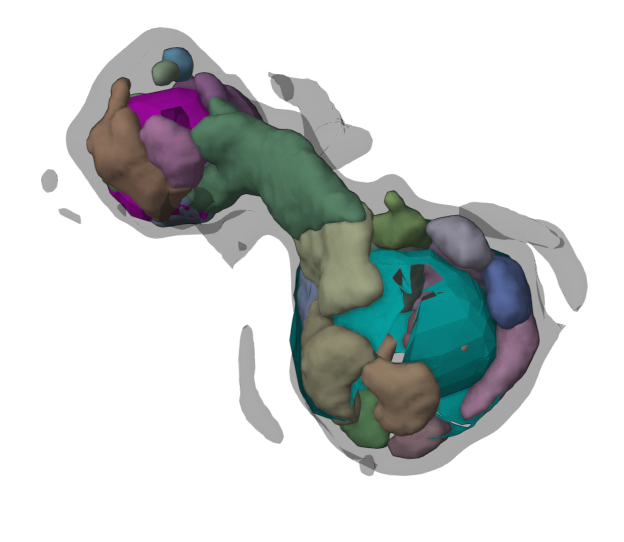

Supplement: gkad411_Supplemental_Files [file gkad411_supplemental_files.zip › molstar-volseg-master/frontend/public/img/examples/emd-9094.png]

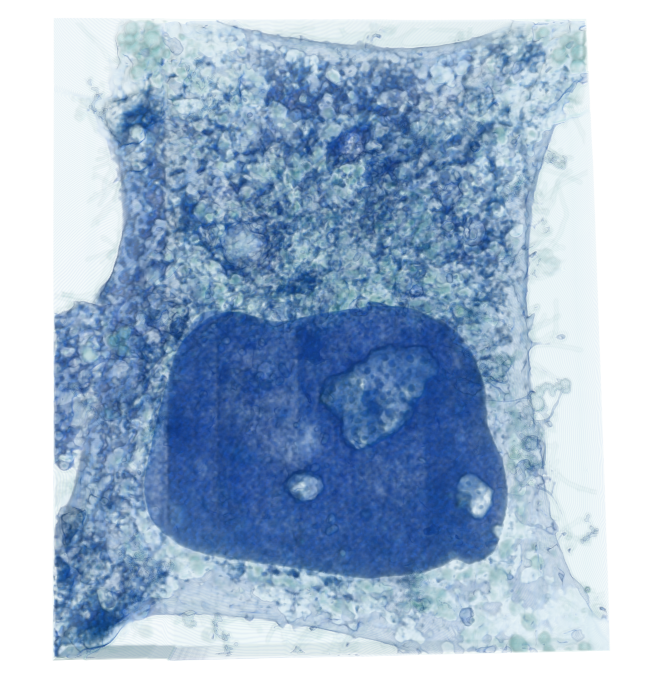

Supplement: gkad411_Supplemental_Files [file gkad411_supplemental_files.zip › molstar-volseg-master/frontend/public/img/examples/empiar-10819.png]

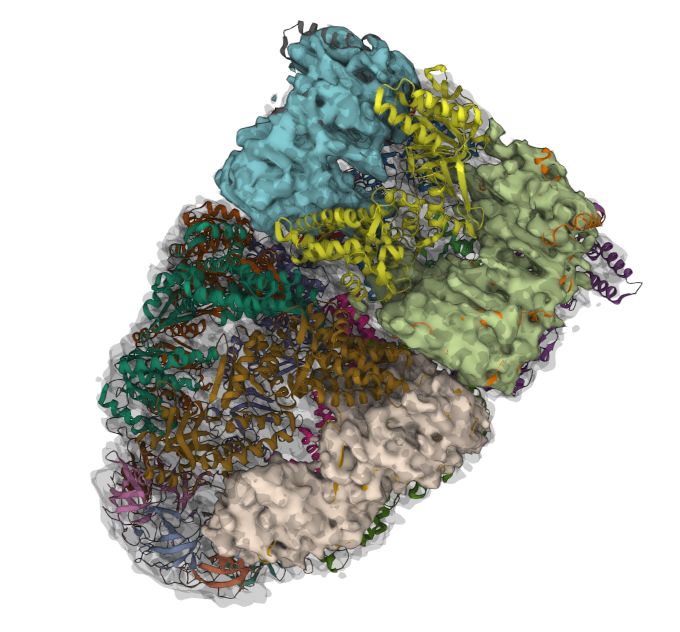

Supplement: gkad411_Supplemental_Files [file gkad411_supplemental_files.zip › molstar-volseg-master/frontend/public/img/examples/ex-emd-1181.png]

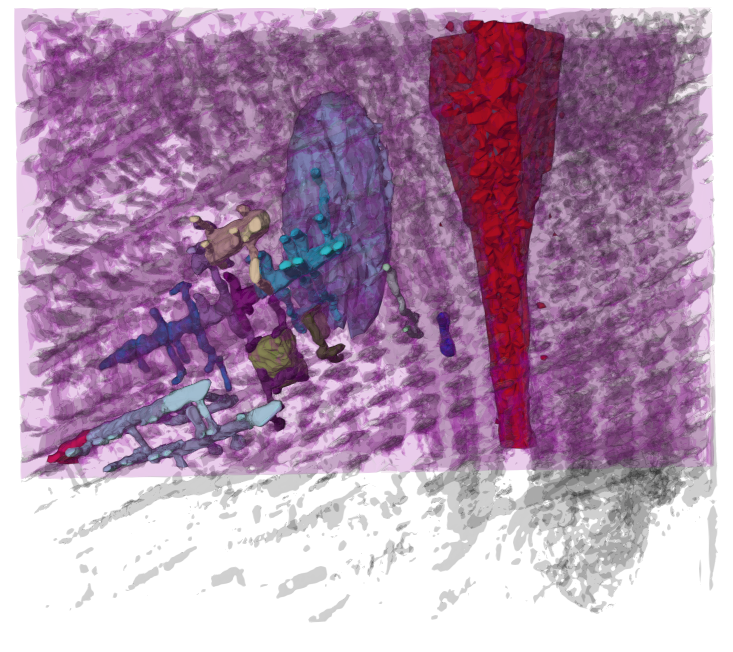

Supplement: gkad411_Supplemental_Files [file gkad411_supplemental_files.zip › molstar-volseg-master/frontend/public/img/examples/ex-empiar-10070.png]

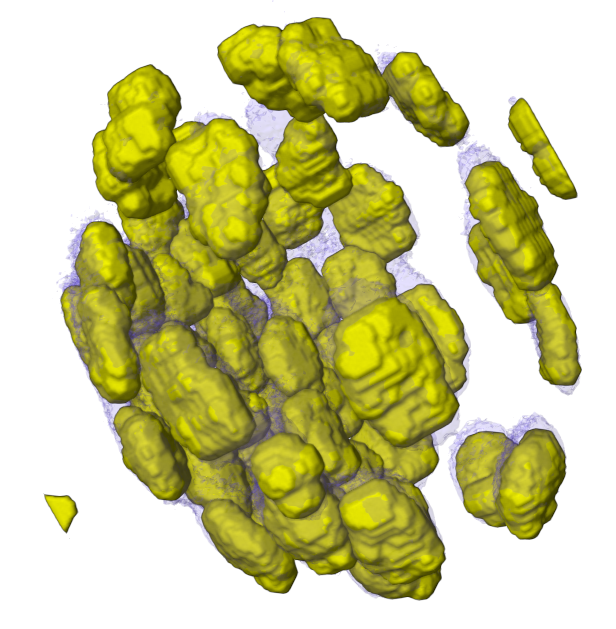

Supplement: gkad411_Supplemental_Files [file gkad411_supplemental_files.zip › molstar-volseg-master/frontend/public/img/examples/ex-idr-6001240.png]

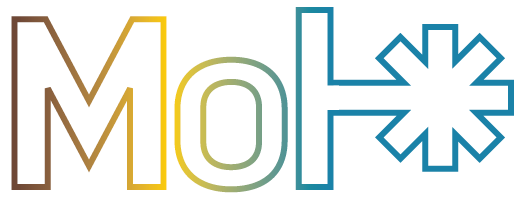

Supplement: gkad411_Supplemental_Files [file gkad411_supplemental_files.zip › molstar-volseg-master/frontend/public/img/molstar-logo.png]

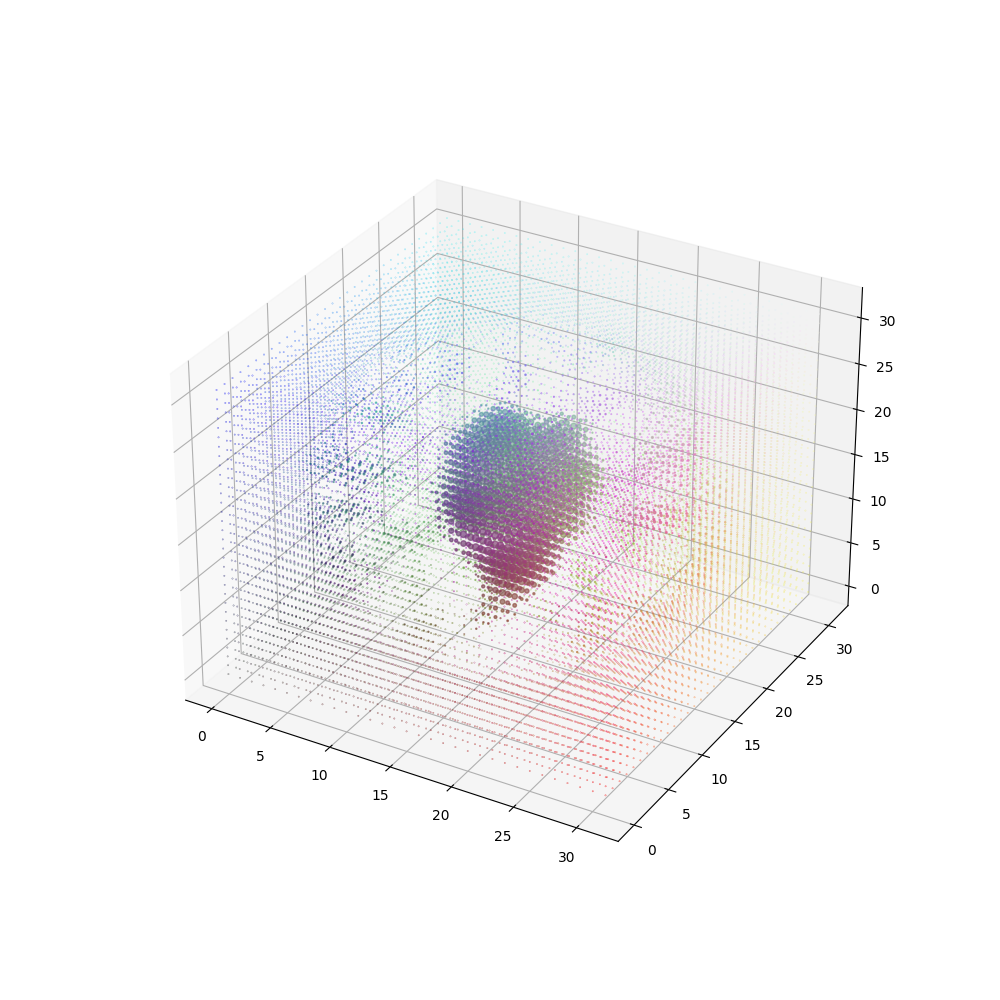

Supplement: gkad411_Supplemental_Files [file gkad411_supplemental_files.zip › molstar-volseg-master/preprocessor/tests/sample_arr_plots/average_2x2x2_blocks_scipy.ndimage.convolve_constant_(32, 32, 32)-grid_adjust_then_negative_to_zero.png]

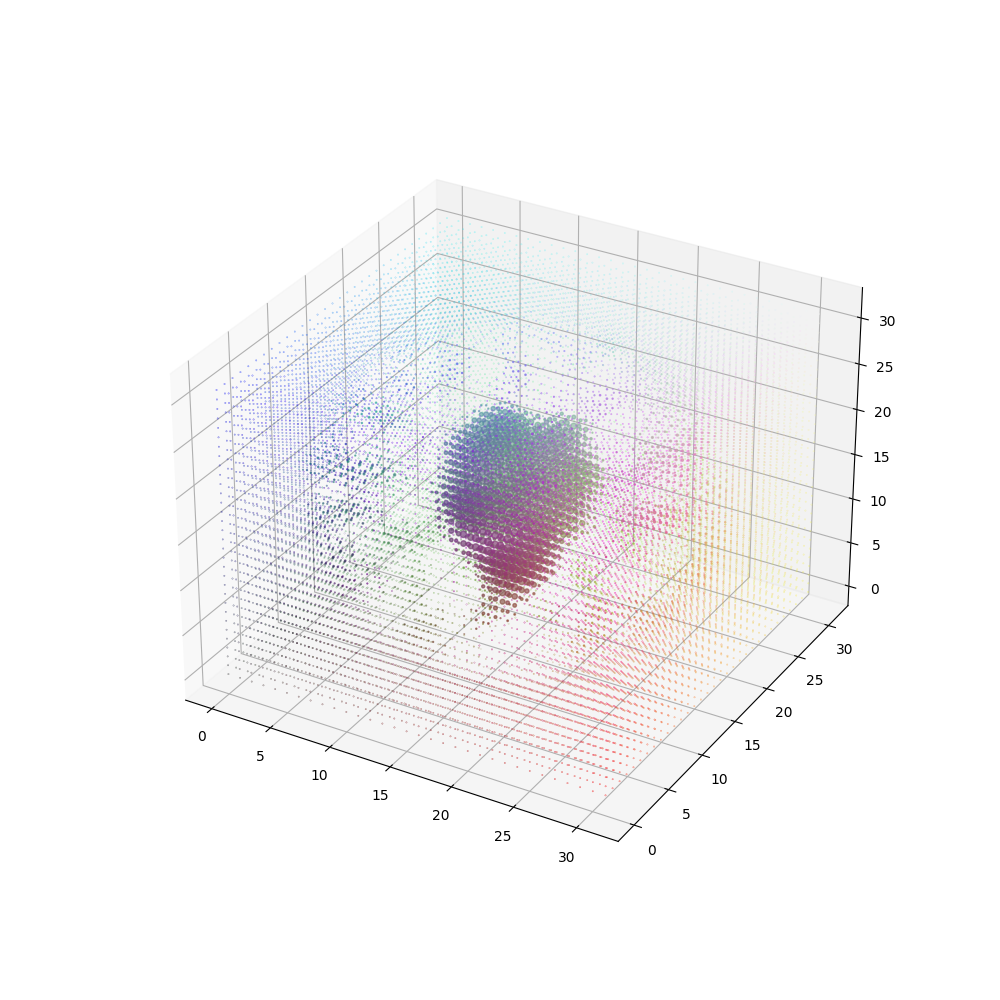

Supplement: gkad411_Supplemental_Files [file gkad411_supplemental_files.zip › molstar-volseg-master/preprocessor/tests/sample_arr_plots/average_2x2x2_blocks_scipy.ndimage.convolve_mirror_(32, 32, 32)-grid_adjust_then_negative_to_zero.png]

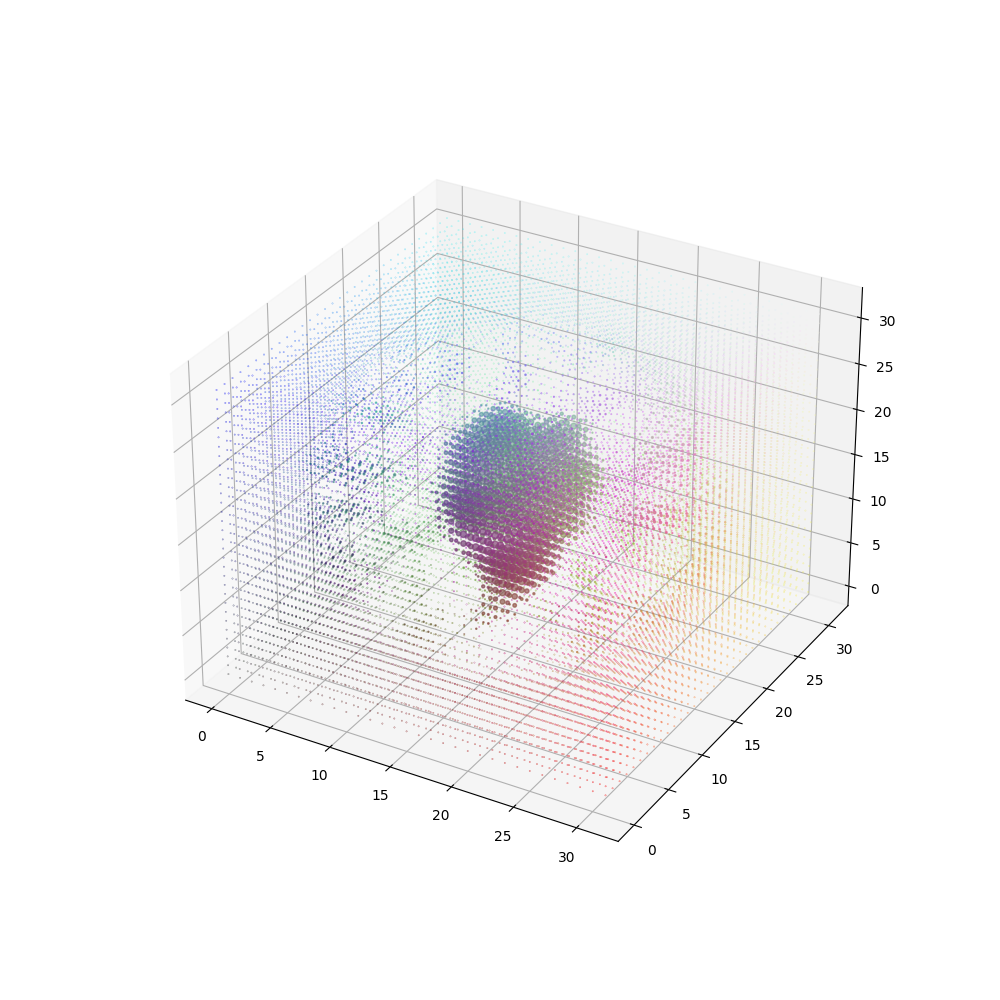

Supplement: gkad411_Supplemental_Files [file gkad411_supplemental_files.zip › molstar-volseg-master/preprocessor/tests/sample_arr_plots/average_2x2x2_blocks_scipy.ndimage.convolve_reflect_(32, 32, 32)-grid_adjust_then_negative_to_zero.png]

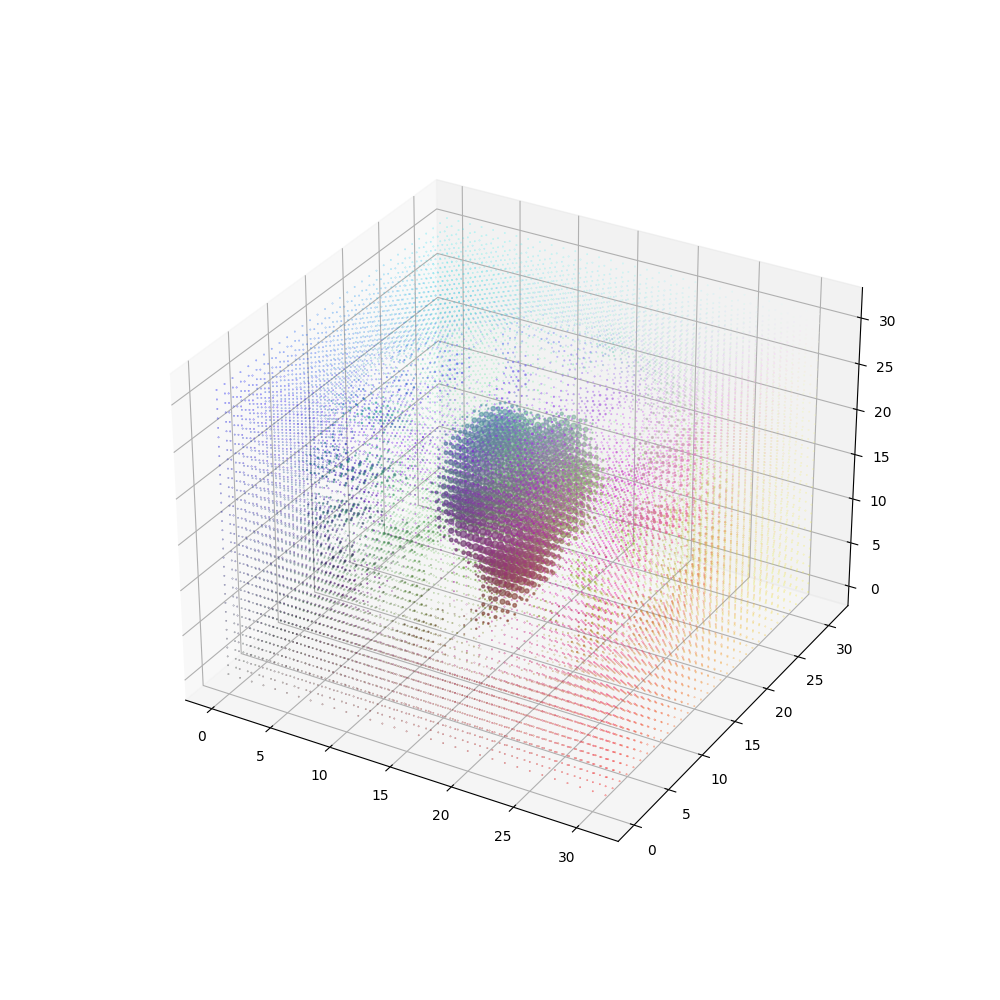

Supplement: gkad411_Supplemental_Files [file gkad411_supplemental_files.zip › molstar-volseg-master/preprocessor/tests/sample_arr_plots/average_2x2x2_blocks_scipy.signal.convolve_fft_(32, 32, 32)-grid_adjust_then_negative_to_zero.png]

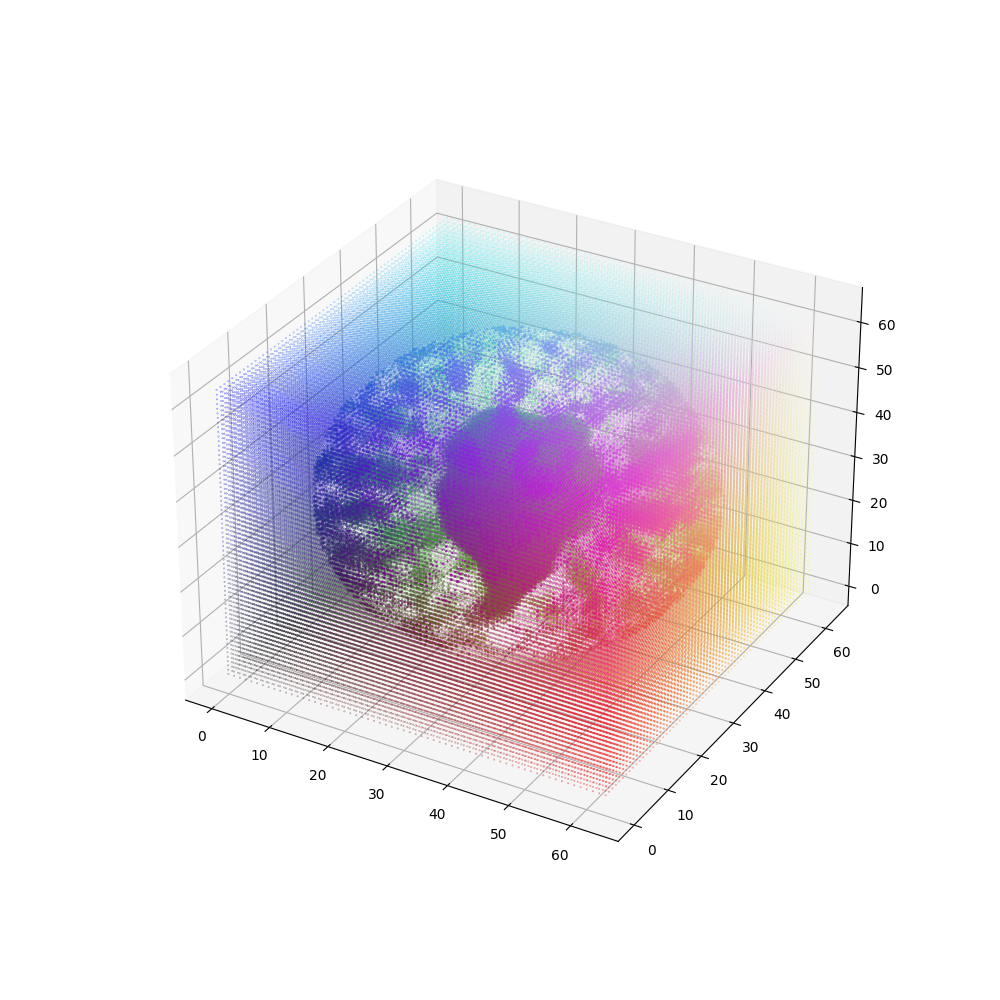

Supplement: gkad411_Supplemental_Files [file gkad411_supplemental_files.zip › molstar-volseg-master/preprocessor/tests/sample_arr_plots/original_(64, 64, 64)-grid_adjust_then_negative_to_zero.png]

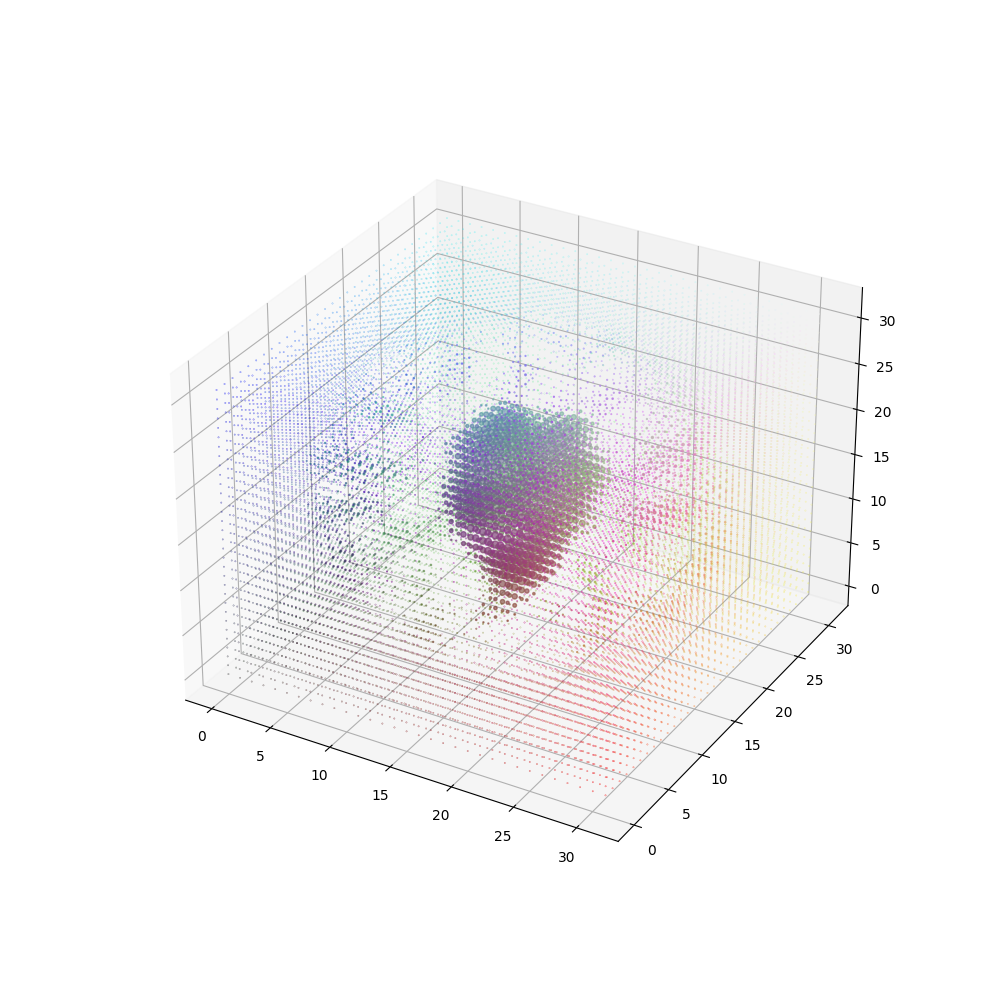

Supplement: gkad411_Supplemental_Files [file gkad411_supplemental_files.zip › molstar-volseg-master/preprocessor/tests/sample_arr_plots/regular_scipy.ndimage.convolve_constant_(32, 32, 32)-grid_adjust_then_negative_to_zero.png]

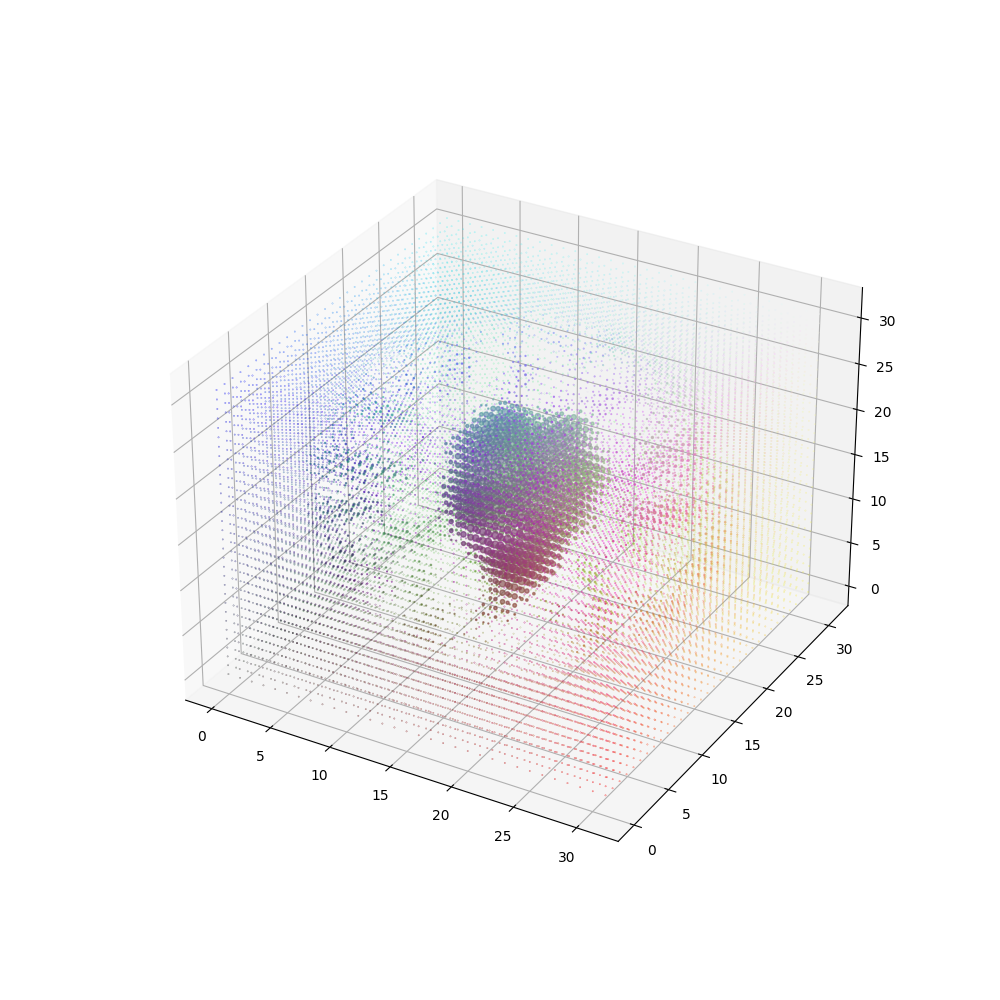

Supplement: gkad411_Supplemental_Files [file gkad411_supplemental_files.zip › molstar-volseg-master/preprocessor/tests/sample_arr_plots/regular_scipy.ndimage.convolve_mirror_(32, 32, 32)-grid_adjust_then_negative_to_zero.png]

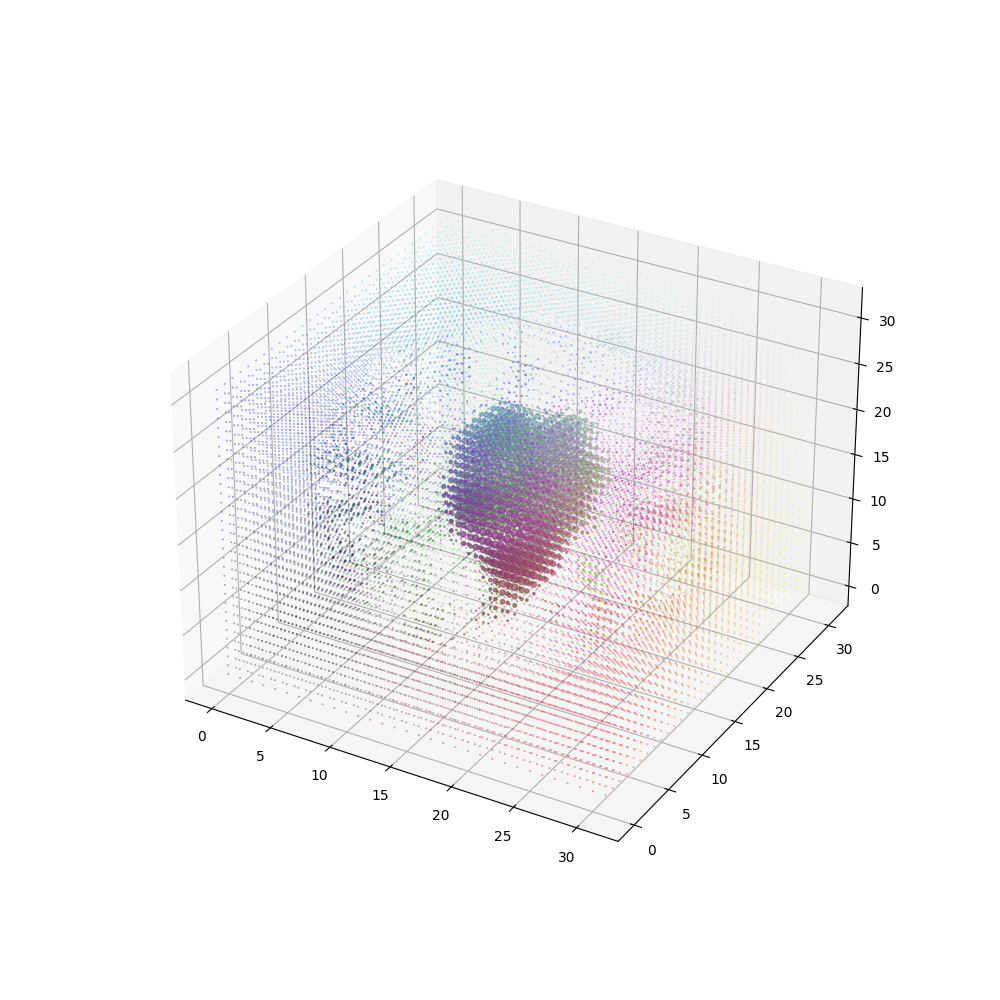

Supplement: gkad411_Supplemental_Files [file gkad411_supplemental_files.zip › molstar-volseg-master/preprocessor/tests/sample_arr_plots/regular_scipy.ndimage.convolve_reflect_(32, 32, 32)-grid_adjust_then_negative_to_zero.png]

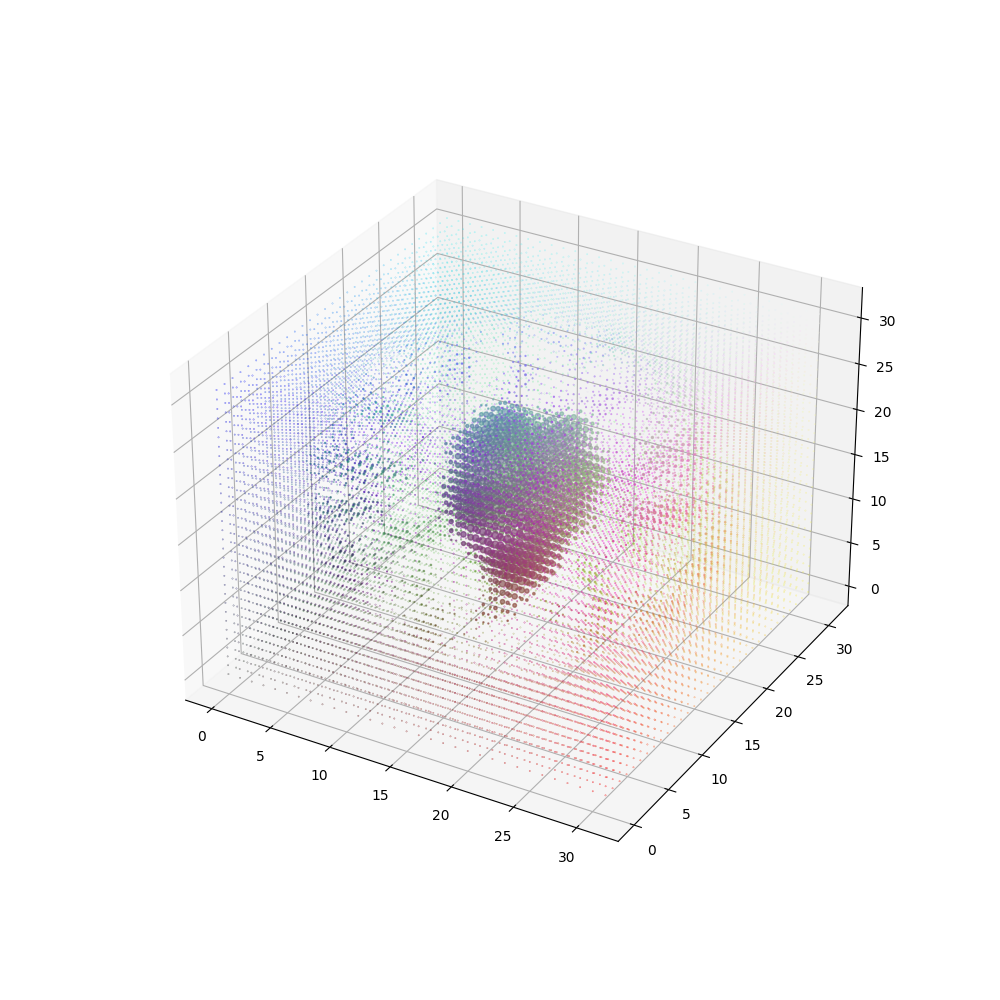

Supplement: gkad411_Supplemental_Files [file gkad411_supplemental_files.zip › molstar-volseg-master/preprocessor/tests/sample_arr_plots/regular_scipy.signal.convolve_fft_(32, 32, 32)-grid_adjust_then_negative_to_zero.png]
